# How multiple causes combine: independence constraints on causal inference

**DOI:** 10.3389/fpsyg.2015.01135

**Published:** 2015-08-10

**Authors:** Mimi Liljeholm

**Affiliations:** Department of Cognitive Sciences, University of CaliforniaIrvine, CA, USA

**Keywords:** causal power, confounding, interaction, uncertainty, Bayesian inference

## Abstract

According to the *causal power view, two* core constraints—that causes occur independently (i.e., no confounding) and influence their effects independently—serve as boundary conditions for causal induction. This study investigated how violations of these constraints modulate uncertainty about the existence and strength of a causal relationship. Participants were presented with pairs of candidate causes that were either confounded or not, and that either interacted or exerted their influences independently. Consistent with the causal power view, uncertainty about the existence and strength of causal relationships was greater when causes were confounded or interacted than when unconfounded and acting independently. An elemental Bayesian causal model captured differences in uncertainty due to confounding but not those due to an interaction. Implications of distinct sources of uncertainty for the selection of contingency information and causal generalization are discussed.

## Introduction

How do multiple causes combine to influence their effects? According to the *causal power view* (Cartwright, 1989; Cheng, 1997), learners apply a set of generic, *a priori*, constraints that enable the “teasing apart” of individual causal influences. Unlike models that simply assess co-variation between putative causes and effects (e.g., Jenkins and Ward, 1965; Rescorla and Wagner, 1972; Van Hamme and Wasserman, 1994), the causal power view explains observed co-variation by postulating underlying, unobservable, causal capacities. Two core constraints of the causal approach—that candidate causes occur independently (i.e., no confounding) and influence their effects independently—serve as boundary conditions for estimating causal strength^1^. This article examines how human reasoners react when the constraints on causal inference specified by the causal power view are violated.

As an illustration, consider three scenarios involving a target candidate cause, X, an alternative cause, Y, and the presence (+) or absence (−) of some relevant effect. In the first scenario, the effect occurs when X and Y are presented in combination, but not when Y is presented alone [XY+, Y−]. In the second scenario, the Y− trials are removed [XY+]. In the third scenario, X− trials are added to the first scenario [XY+, Y−, X−]. How would judgments about X differ across these three scenarios? According to the causal power approach, it is only in the first scenario that the individual influence of X can be teased apart from that of Y: In the second scenario X is confounded with Y, violating the^1^ “no confounding” constraint, and in the third scenario, X and Y interact, violating the assumption of independent causal influence.

A probabilistic formalization of the causal power view, the power-PC theory (Cheng, 1997), mathematically defines the problem with estimating the strength of confounded causes: Whenever the probability of the occurrence of one candidate cause (e.g., Y) differs across the absence and presence of another (e.g., X), the power-PC equations contain multiple unknowns so that there is no unique solution. Thus, “no confounding” as a constraint is a result of the power-PC theory (see Cheng, 1997 for details). Although consistent with data showing sensitivity to confounding in the selection of frequency information, estimates of outcome probabilities, exploratory behavior and causal model selection (Spellman, 1996; Kushnir and Gopnik, 2005; Meder et al., 2006; Schulz et al., 2007; Schulz and Bonawitz, 2007), there is also ample evidence that subjects are willing to provide a point-estimate of causal strength when asked to do so, even under confounded conditions (Wasserman and Berglan, 1998; Waldmann, 2001; Lovibond et al., 2003; Beckers et al., 2005; Le Pelley et al., 2005). One possibility is that subjects employ some heuristic (e.g., averaging across possible causal strengths) when asked to give a single estimate but that, due to the lack of a unique solution, such estimates are in fact associated with high levels of uncertainty.

Novick and Cheng (2004) extended the power-PC theory to the case of interacting, or *conjunctive*, causes arguing that, as with simple causal powers, the discovery of conjunctive powers requires postulating the existence of unobservable causal capacities. While it successfully accounts for conditions under which reasoners infer causal interactions (Novick and Cheng, 2004; Liljeholm and Cheng, 2007; Liljeholm et al., 2007), the conjunctive power theory is silent on the issue of corollary increases in uncertainty about simple causal powers. Consider again the third scenario described above, in which neither X nor Y generate the effect individually but only do so when combined. Here, according to the conjunctive power theory, reasoners will infer the existence of a conjunctive node “XY” and will continue to assume that all distinct causes, X, Y, and XY, influence the effect independently of one another, as well as of any other candidate causes. However, given that candidate X (and/or Y) has been found to violate the default assumption of independent influence—a fundamental constraint on causal inference—one might expect that further induction about the influence exerted by X, particularly in novel contexts and compounds, would be fraught with uncertainty.

Although the causal power view implies that interacting and confounded causes should elicit high levels of uncertainty, neither the power-PC theory nor the conjunctive power theory provides an explicit, quantitative, measure of uncertainty. Such a measure is provided, however, by the framework of Bayesian causal models (Tenenbaum and Griffiths, 2001; Griffiths and Tenenbaum, 2005; Lu et al., 2008). According to Bayesian models of elemental causal induction, involving a single candidate cause and a constant background cause, reasoners decide whether a set of observations (*D*) was generated by a causal graphical structure in which a link exists between the candidate cause *c* and effect *e* (Graph 1) or by a causal structure in which no link exists between *c* and *e* (Graph 0); both graphs assume the existence of a link between background cause *b* and *e*. When the prior probabilities of the two graphs are equal, the decision variable, termed “Causal support,” is the log ratio of the likelihoods of the data (*D*) given Graphs 1 and 0.

(1) $$\begin{array}{l} log\frac{P\left(D|Graph1\right)}{P\left(D|Graph0\right)} \end{array}$$

The likelihoods are derived using a particular parameterization that specifies how causes combine to influence their effects. For example, the parameterization adopted by the power-PC theory, and following from its assumptions (Cheng, 1997; Glymour, 1998), is the noisy-OR:

(2) $$\begin{array}{l} P\left(e^{+}|b,c;w_{B},w_{C}\right)={w_{B}}^{b}+{w_{C}}^{c}-{w_{B}w_{C}}^{bc} \end{array}$$

where *c* is 0 or 1 depending on the absence vs. presence of the candidate cause, *b* is always 1 (i.e., present), and *w*_*B*_ and *w*_*C*_ are parameters associated with the strength of the background and candidate cause, respectively. Causal power is a maximum likelihood estimator of *w*_*C*_ under this parameterization.

Critically, in the Bayesian framework, the posterior distribution on *w*_*C*_ provides a basis for distinguishing between strength and uncertainty about strength: Whereas the location of the peak of this distribution is the maximum a posteriori estimate of causal strength, the degree of uniformity of the distribution indicates uncertainty in that estimate (Griffiths and Tenenbaum, 2005). For example, in the most extreme case, when the distribution is uniform, all possible values of strength are equally likely and uncertainty about the “true” strength is maximal. Thus, this model provides an explicit representation of uncertainty in strength estimates, as well as uncertainty about the existence of a causal relationship. The present study extends Griffiths and Tenenbaum's (Tenenbaum and Griffiths, 2001; Griffiths and Tenenbaum, 2005) Bayesian causal model to the case of two candidate causes and uses this extension as a normative framework for assessing how violations of causal power assumptions influence uncertainty in causal inference.

## Methods

### Participants

One hundred and eight undergraduates at the University of California, Los Angeles participated in the study to obtain course credit. All participants gave informed consent and the study was approved by the Institutional Review Board of the University of California, Los Angeles.

### Design

The study focused on two target causes, C and I, mnemonically labeled here to indicate confounded and interacting causes, respectively^2^. Each target cause occurred in one of two non-overlapping compounds, with one compound (CD) consisting of confounded causes, and the other (IJ) of interacting causes. Specifically, in the first phase, candidates C and D *only* occurred in combination, such that the two causes were perfectly confounded. Then, in a second phase, trials were introduced in which candidate C occurred without D, thus unconfounding the influences of the two causes. The causal power approach suggests that uncertainty about the existence and strength of a causal relationship between candidate C and the effect should be greater in the first phase than the second. While candidate I likewise only occurred together with its compound counterpart (J) in Phase 1, candidate J also occurred in the absence of I in this phase, enabling an estimation of the independent causal influences of the two causes. However, in the second phase, additional trials in which I occurred without J suggested that the two causes acted differently in compound than what would be expected based on their individual influences (i.e., I and J apparently interacted). Given this violation of the assumption of independent causal influence, the causal power approach suggests that uncertainty about the causal status of candidate I should be greater in the second phase than in the first.

According to the causal power view, if a binary effect always occurs in the presence of some candidate cause (e.g., C), the capacity of an alternative cause (e.g., D) to exert generative influence will be occluded. Consequently, such a “ceiling effect” constitutes yet another violation of the boundary conditions for computing causal strength (Cheng, 1997). Here, to restrict scenarios to violations of the “no confounding” and “independent influence” constraints, the probability of the effect in the presence of any given cause, or compound of causes, was always less than 1.0. Specifically, the probability of the effect in the presence of the CD compound was 0.8, rather than 1.0, thus avoiding the ceiling effect. The probabilities of the effect in the presence of candidate J and the IJ compound were such that the increase in the probability of the effect when candidate I was added to candidate J was one half (0.4) of the probability of the effect on CD trials: If candidates C and D are assumed to contribute equally, and linearly (Rescorla and Wagner, 1972) to the probability of the effect on CD trials, this equates the strengths of candidates I and C in the first phase. Finally, to assess the generality of obtained differences in uncertainty across different levels of causal strength, the probabilities of the effect in the presence of target candidates C and I in the second phase were either high (0.8) or low (0.0).

The different conditional probabilities of the effect were implemented in two groups, respectively labeled “C_strong__I_weak_” and “C_weak__I_strong_,” with subscripts “strong” and “weak” indicating the model-derived strength of the relevant target cause by the end of the second phase. In group C_strong__I_weak_, the probability of the effect in the presence of candidate C in the second phase was 0.8, while that in the presence of candidate I was 0.0. Conversely, in group C_weak__I_strong_, the probability of the effect in the presence of candidate C in the second phase was 0.0, while that for candidate I was 0.8. Importantly, in group C_weak__I_strong_, in which the probability of the effect in the presence of candidate I in the second phase was high (0.8), the effect probabilities in the presence of candidate J and compound IJ were also greater (0.4 and 0.8, respectively) than those in group C_strong__I_weak_. This was done because, had these probabilities remained at 0.0 and 0.4, respectively (as in group C_strong__I_weak_), candidate J would be considered an inhibitory, or preventive, cause given the conditional probabilities in the second phase; in other words, there would be no violation of the assumption of independent causal influence. Participants were randomly assigned to the two groups. The frequencies of all trial types, for each group and each phase, are shown in Table 1.

**Table 1:** **Experimental design**.

**Trial types**	**C_strong__I_weak_**		**C_weak__I_strong_**	
	**Phase 1**	**Phase 2**	**Phase 1**	**Phase 2**
CD+, CD−	16, 4	16, 4	16, 4	16, 4
C+, C−	0,0	16,4	0,0	0,20
IJ+, IJ−	8,12	8,12	16,4	16,4
I+, I−	0,0	0,20	0,0	16,4
J+, J−	0,20	0,20	8,12	8,12
NM+, NM−	0,20	0,20	0,20	0,20

*Frequencies of trial types, shown in the leftmost column, for each group and phase. The first and second entry in each cell indicates the presence (+) and absence (−) of the effect, respectively. NM, No medicine*.

At the end of each phase, participants were asked to select between the following options regarding the influence of each target candidate causes: “produces the effect,” “has no influence on the effect” and “can't tell.” The proportion of “can't tell” choices was used as a measure of uncertainty about the existence of a causal relationship. Participants were then asked, regardless of their answer to the first query, to rate the strength of the influence of the candidate cause on a scale from 0 (not at all strongly) to 100 (extremely strongly) and, finally, to indicate how confident they were in their estimate of strength, again on a scale from 0 (not at all confident) to 100 (extremely confident).

### Materials and procedure

**Figure 1:**
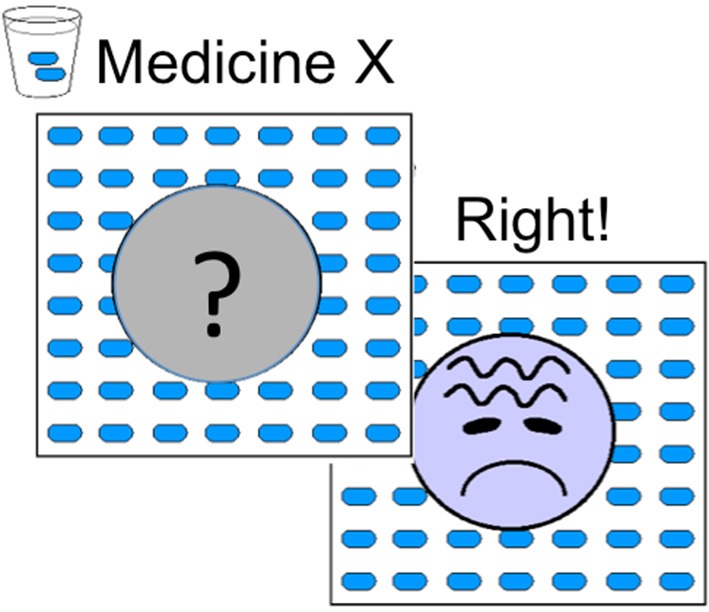
**Trial illustration**. On each trial, participants saw a screen indicating which medicine(s), or that no medicine, had been ingested, with the face indicating the allergy patients state being covered by a gray circle and a question mark. They were asked if the allergy patient had a headache, and responded yes or no by pressing the “Y” and “N” keys, respectively. The subsequent screen showed the allergy patient with headache or without headache (indicated by a smiley face) together with feedback about whether the participant was correct or not.

The stimuli were presented on a computer and booklets were provided for writing down causal judgments. At the beginning of the experiment, participants were given a cover story informing them that they would play the role of a research scientist assessing the influences of various allergy medicines on headache, a potential side effect. They were told that each medicine could either produce headache or have no influence on headache (i.e., there were no preventive causes). On each trial (see Figure 1), participants were presented with a picture of an individual allergy patient and were told either that the patient had not received any medicine or that a particular medicine, or combination of medicines, had been administered to the patient. On the first screen, the allergy patients' face was covered with a gray circle with a question mark printed on it and participants were asked to predict whether or not the patient had a headache given the administered medicine(s), by pressing the “Y” key to indicate “yes” or the “N” key to indicate “no.” After making a prediction, subjects were given feedback, which consisted of a picture indicating the allergy patient's state (i.e., with or without headache) and a statement about whether the subject's prediction was right or wrong. None of the allergy patients had headache in the absence of any medicine and this was explicitly stated in the initial instructions, as well as apparent on 20 “No Medicine” trials presented in each phase. Each type of medicine(s), and no medicine, trial within a group was presented 20 times in each phase in which the type occurred, for a total of 200 trials (see Table 1).

Prior to viewing any data, participants were told that, after observing the data, they would be required to answer questions about the influences of the medicines on headache and that, therefore, they needed to pay close attention to the feedback on all trials. At the end of the first phase, participants were asked the following questions about each medicine:

1. Based on ALL of the information you've seen so far, what is the overall influence of this medicine?
  - This medicine produces headache
  - This medicine has no influence on headache
  - Can't tell
2. Regardless of your answer to question 1, based on ALL of the information you've seen so far, what is your best estimate of the overall strength of the influence of this medicine on headache?
3. How confident are you in your estimate of strength provided above?

The emphasis on “ALL of the information” and “overall influence/strength” was intended to highlight the importance of generalizable causal knowledge. It is, of course, trivial to estimate the probability with which the effect occurs when candidate I is presented without candidate J in the second phase. However, given that candidate I acts differently when presented in compound with J than when presented individually, it is difficult to predict how this candidate would act if presented in a novel compound.

Strength estimates and confidence in those estimates were rated on scales ranging from 0 (not at all) to 100 (extremely), as described above. Rating scales were printed with extreme numbers at respective endpoints and with numerical labels indicating increments of 10. After answering the queries following the first phase, participants were told that they would be shown more data from tests of the medicines, and that they would subsequently be asked questions about causal influences again. Finally, following the second phase, participants were again presented with the three queries regarding structure, strength, and uncertainty about strength.

At the end of each phase, for each medicine queried, a summarized display of the trials from that phase, relevant for evaluating the particular medicine, was shown on the screen together with instructions to turn to the next page of the response booklet, to write down the identity of the particular medicine at the top of that page, and to answer the questions about that medicine, all three of which were printed on each page of the booklet. While ratings were collected for all causes following the first phase, to avoid biasing subsequent learning, only target causes (C and I) were queried after the second phase, with the script terminating following responses to the second target cause. The order of causes being queried at the end of each phase was random.

### Bayesian causal model

**Figure 2:**
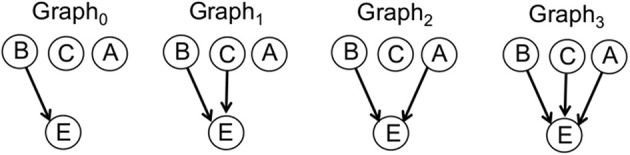
**Causal graphs**. Explanation of the data observed by participants as causal graphs in which a causal influence over the effect (E), indicated by an arrow, exists for neither the target cause (C) nor the alternative (A) cause (Graph0), for the target cause but not the alternative cause (Graph1), for the alternative cause but not the target cause (Graph2), or for both candidate causes (Graph3).

The causal support model with uniform priors (Tenenbaum and Griffiths, 2001; Griffiths and Tenenbaum, 2005) was extended to the case of two candidate causes and a constant background, such that the hypothesis space consisted of four graphs, all of which include a causal link from the background to the effect. A full account of the Bayesian causal model is presented in Appendix. For generality, all subsequent model equations, in the main text and in the Appendix, will use *C* to indicate the target cause, *A* to indicate the alternative candidate cause and *B* to indicate the constant background. The four graphs making up the hypothesis space are illustrated in Figure 2: In the first graph, only the background (B) has a link to the effect (Graph0); in the second graph, the target cause (C) has a link to the effect, but the alternative candidate cause (A) does not (Graph1); in the third graph, the alternative candidate cause, but not the target cause, has a link to the effect (Graph2); in the fourth graph, both candidate causes have links to the effect (Graph3). We only consider generative causes, since participants were explicitly instructed that candidate causes either produced the effect or had no influence. The model was instantiated in each of four scenarios, (i.e., for each condition, confounding and interaction, and in each phase), with predictions derived for the target cause in each scenario. Specifically, for each target cause, causal support was defined as the ratio of likelihoods on graphs in which a link exists between the target cause and the effect over graphs in which no link exists for the target cause:

(3) $$\begin{array}{l} \sup_{C\to E}=\log\frac{P\left(D|Graph1\right)+P\left(D|Graph3\right)}{P\left(D|Graph0\right)+P\left(D|Graph2\right)} \end{array}$$

The likelihoods were computed by averaging out the unknown causal strengths (*w*_*i*_) of the background, target cause and alternative candidate cause such that, for example, for Graph3:

(4) $$\begin{array}{l} P(D|Graph3)=\int_{0}^{1}\int_{0}^{1}\int_{0}^{1}P(D|w_{B},w_{C},w_{A},Graph3)\\ \;P(w_{B},w_{C},w_{A}|Graph3)dw_{B}dw_{C}dw_{A} \end{array}$$

where the likelihood terms are derived using a causal power parameterization^3^ (see Equation 2).

A continuous measure of confidence in the existence of a causal link (Tenenbaum and Griffiths, 2001; Griffiths and Tenenbaum, 2005), causal support is strongly positive if a causal relationship is very likely, strongly negative if a causal relationship is very unlikely, and close to zero when the status of a causal relationship is unknown. Behavioral “can't tell” choices were coded as zero, “produces” choices as 1, and the “no influence” choices as −1, and mean judgments were then modeled as causal support. Following Griffiths and Tenenbaum (2005), a scaling transformation was applied to causal support values. The posterior distribution on *w*_*C*_ in the most likely graph in which a link existed between the target cause and the effect (i.e., Graph1 or Graph3, depending on which had the highest likelihood) was used to model strength estimates and uncertainty in strength estimates. Following Lu et al. (2008), strength estimates were modeled as the mean of *w*_*C*_:

(5) $${\bar{w}}_{C}=\int_{0}^{1}w_{C}P(w_{C}|D)dw_{C}.$$

Uncertainty in strength was quantitatively defined as the Shannon entropy of the posterior distribution on *w*_*C*_, *H(w*_*C*_*)*, which is greatest when the distribution is uniform (i.e., all strengths are equally probable):

(6) $$H(w_{C})=-\int_{0}^{1}P(w_{C}|D)\ln P(w_{C}|D)dw_{C}.$$

To equate the scale with strength ratings, model-derived strength estimates were multiplied by 100. Likewise, since Shannon entropy reflects the degree of uncertainty, while participants rated confidence, or certainty, mean confidence ratings were subtracted from 100 to equate the directions of the scales.

## Results

**Figure 3:**
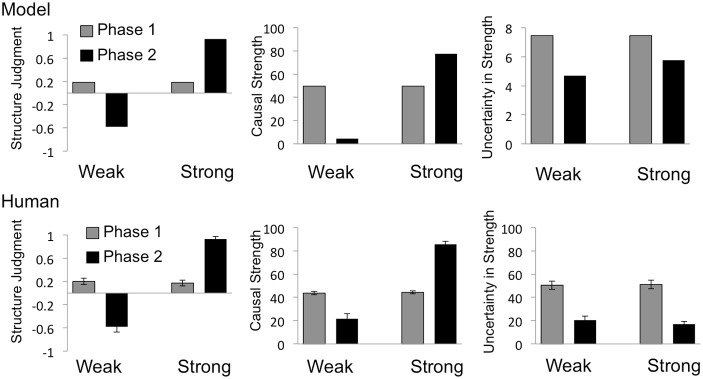
**Model predictions and behavioral results for the confounded target cause**. Predictions of the Bayesian causal model are shown in the top row, and mean human judgments in the bottom row, for structure judgments (left), strength estimates (middle) and uncertainty in strength estimates (right). Structure judgments were derived by coding structure choices as “No influence” = −1, “Can't tell” = 0, “Generative influence” = 1. Labels “Weak” and “Strong” indicate the model-derived strength of the target cause (C) at the end of the 2nd phase. Error bars = SEM.

**Figure 4:**
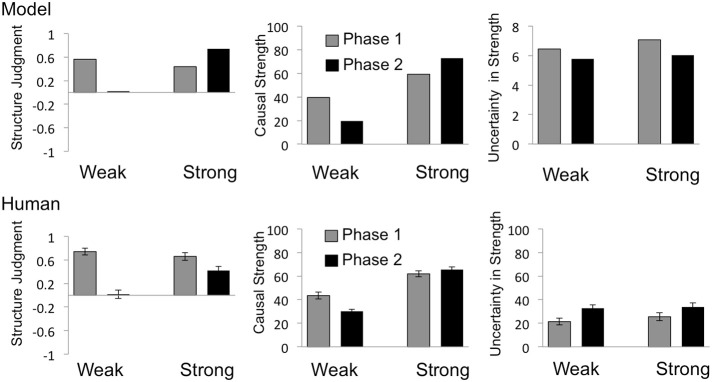
**Model predictions and behavioral results for the interacting target cause**. Predictions of the Bayesian causal model are shown in the top row, and mean human judgments in the bottom row, for structure judgments (left), strength estimates (middle) and uncertainty in strength estimates (right). Structure judgments were derived by coding structure choices as “No influence” = −1, “Can't tell” = 0, “Generative influence” = 1. Labels “Weak” and “Strong” indicate the model-derived strength of the target cause (I) at the end of the 2nd phase. Error bars = SEM.

Human data for structure, strength and uncertainty about strength are shown in the bottom rows of Figure 3 (for the confounded target cause) and Figure 4 (for the interacting target cause), with corresponding plots of model prediction shown in the top rows. A 2 (Phase) × 2 (Group) mixed analysis of variance (ANOVA) was performed on each type of rating (strength and uncertainty about strength) and for each type of causal power violation (confounding and interaction). The results of these analyses are reported in relevant subsections. Cohen's *d*_*z*_ (hereafter *d*_*z*_) is reported for all pairwise comparisons. Throughout the results, group-labels “Strong” and “Weak” refer to the model-derived strength of the relevant target cause at the end of Phase 2.

### Confounded causes

#### Structure judgments

Consistent with the causal power view, as can be seen in Figure 3, both human judgments and the Bayesian causal model reflect high levels of uncertainty associated with confounded target cause C in Phase 1; uncertainty that is subsequently resolved by individual presentations of that cause either with or without the effect in Phase 2.

Specifically, when asked to categorically judge the influence of a confounded candidate cause, at the end of the first phase, 88 out of 108 participants chose the “can't tell” option, suggesting low confidence in their ability to evaluate the existence of a causal link. After viewing the data from Phase 2, in which the relevant cause was presented without the confounding alternative cause, participants in the Strong group overwhelmingly chose the “produces” option (54 out of 58), while those in the Weak group chose the “no influence” option (35 out of 50). Chi square tests performed on these judgments revealed that the proportion of “can't tell” choices decreased significantly from the first to the second phase in both the Strong (χ^2^ = 70.96, *n* = 58, *p* < 0.001) and Weak (χ^2^ = 55.61, *n* = 50, *p* < 0.001) group. Consistent with this pattern of results, causal support for the relevant candidate cause starts out close to zero at the end of the first phase, but becomes strongly positive and strongly negative, for the Strong and Weak group respectively, at the end of Phase 2.

#### Strength ratings

A 2 (Phase) × 2 (Group) ANOVA performed on strength ratings for the confounded target cause revealed a significant main effect of Phase, *F*_(1, 106)_ = 18.44, *p* < 0.001, a significant main effect of Group *F*_(1, 106)_ = 123.30, *p* < 0.001, and a significant Phase by Group interaction, *F*_(1, 106)_ = 129.13, *p* < 0.001. Planned comparisons further revealed that, across the two phases, mean strength ratings increased significantly in the Strong group [from 44.4 to 85.7; *t*_(57)_ = 14.11, *p* < 0.001, *d*_*z*_ = 0.63] and decreased significantly in the Weak group [from 43.70 to 21.60; *t*_(49)_ = 4.46, *p* < 0.001, *d*_*z*_ = 1.82]. Importantly, as can be seen in Figure 3, in spite of the high levels of uncertainty associated with the confounded cause at the end of the first phase, mean ratings of its causal strength were as predicted by the Bayesian causal model. In contrast, according to the power-PC theory, no unique solution for causal strength exists at this point. These results suggest that strength ratings do not necessarily reflect violations of causal power assumptions even when ratings of uncertainty do: a fact that may have obfuscated the use of causal power assumptions in studies of human causal learning that do not measure uncertainty. The Bayesian causal model also predicted the increase in strength ratings across phases in the Strong group and decrease across phases in the Weak group.

#### Confidence in strength

When specifically asked about their confidence in strength estimates, it is again apparent that participants were sensitive to a violation of the “no confounding” constraint specified by the causal power approach. A 2 (Phase) × 2 (Group) ANOVA performed on confidence ratings for the confounded target cause revealed a significant main effect of Phase, *F*_(1, 106)_ = 106.69, *p* < 0.001, but no effect of Group *F*_(1, 106)_ = 0.15, *p* = 0.70, nor any interaction, *F*_(1, 106)_ = 0.45, *p* = 0.51. Mean ratings of confidence in strength estimates (bottom right plot in Figure 3) reflect high levels of uncertainty following Phase 1, that are significantly reduced at the end of Phase 2, in both the Strong [from 51.12 to 16.98; *t*_(57)_ = 8.30, *p* < 0.001, *d*_*z*_ = 0.89] and Weak [from 50.50 to 20.50; *t*_(49)_ = 4.46, *p* < 0.001, *d*_*z*_ = 1.09] group. This sensitivity to confounding is ordinally captured by the Bayesian model (top right plot in Figure 3), which predicts high entropy for the posterior distribution over strength after Phase 1, and lower levels of entropy following Phase 2.

### Interacting causes

#### Structure judgments

Consistent with the causal power view, as can be see in the bottom row of Figure 4, human judgments reflect low levels of uncertainty when the target candidate cause exerts an apparently independent influence in Phase 1, and a significant increase in uncertainty when trials were introduced suggesting that candidate causes interact in Phase 2. In contrast, according to the Bayesian model, confidence in the existence of a causal link increases from the first to the second phase for the Strong group (top left plot in Figure 4), while uncertainty about strength decreases across phases in both groups (top right plot in Figure 4).

Specifically, when asked to judge whether or not an apparently independently acting candidate cause had any influence over the effect, at the end of Phase 1, only 30 out of 108 participants chose the “can't tell” option, suggesting high confidence in their ability to evaluate the existence of a causal link. Moreover, out of the remaining 78 participants, 77 indicated that the cause produced the effect, while only one participant choose the “no influence” option. After viewing the data from the second phase, in which the probability of the effect given the relevant causes deviated from that expected based on their individual influences, the majority of participants chose the “can't tell” option in both the Strong (27 out of 50) and Weak (41 out of 58) group. Chi square tests performed on the categorical judgments confirmed that the increase in the proportion of “can't tell” choices from the first to the second phase was significant, in both the Strong (χ^2^ = 6, *n* = 50, *p* < 0.05) and Weak (χ^2^ = 42.30, *n* = 58, *p* < 0.001) group.

Consistent with human judgments in the Weak group, causal support for candidate I was strongly positive based on the data given in the first phase, and then decreased substantially across phases. However, contrary to the pattern of results found for human judgments, causal support *increased* across phases in the Strong group. Note that, since both the individual and combined influence of candidate I was clearly positive in the second phase for the Strong group, one might have expected more participants to choose the “produces” option, consistent with causal support. One possible reason for the proportion of “can't tell” choices in this condition may be that participants postulated that candidate I both (1) generated the effect and (2) prevented the influence of candidate J on the effect, as an explanation for why the occurrence of the effect in the presence of the compound is less than that expected based on independent influences. Regardless, the proportion of “can't tell” choices after the second phase is clearly much greater for the Weak than for the Strong group.

#### Strength ratings

A 2 (Phase) × 2 (Group) ANOVA performed on strength ratings for the interacting target cause (I) revealed a significant main effect of Phase, *F*_(1, 106)_ = 4.96, *p* < 0.05, a significant main effect of Group *F*_(1, 106)_ = 109.50, *p* < 0.001, and a significant Phase by Group interaction, *F*_(1, 106)_ = 11.68, *p* < 0.001. Again, mean causal strength ratings were well accounted for by the Bayesian model (middle column in Figure 4), which accurately predicted the decrease in mean ratings across phases in the Weak group [from 43.54 to 30.17; *t*_(57)_ = 3.76, *p* < 0.005, *d*_*z*_ = 0.49], as well as the increase in the Strong group, from 61.90 to 65.50. Although the increase in strength ratings across phases in the Strong group was not significant (*p* = 0.3), the Bayesian model nicely captures the ordinal pattern of strength ratings across groups and phases.

#### Confidence in strength ratings

As with confidence in strength estimates for the confounded target cause, a 2 (Phase) × 2 (Group) ANOVA performed on confidence ratings for the interacting target cause revealed a significant main effect of Phase, *F*_(1, 106)_ = 23.02, *p* < 0.001, but no effect of Group *F*_(1, 106)_ = 0.42, *p* = 0.52, nor any interaction, *F*_(1, 106)_ = 0.54, *p* = 0.46. Specifically, across the two phases, as the target cause goes from an independent to an interacting influence, mean ratings of uncertainty in strength estimates increased significantly in both the Strong [from 25.50 to 33.80; *t*_(49)_ = 2.45, *p* < 0.05, *d*_*z*_ = 0.35] and Weak [from 21.38 to 32.76; *t*_(57)_ = 4.5, *p* < 0.001, *d*_*z*_ = 0.59] group; in contrast, the Bayesian model predicts a decrease in uncertainty across phases in both groups. Thus, when participants were asked about their confidence in strength estimates, the human data again deviated from the predictions of the Bayesian model (right column in Figure 4).

## Discussion

This study investigated the influence of violations of causal power assumptions on uncertainty about the existence and strength of a causal relationship. Consistent with the causal power approach (Cartwright, 1989; Cheng, 1997), it was found that when a candidate cause was confounded or interacted with another cause, uncertainty in both the existence and strength of a causal relationship was relatively high. In contrast, when candidate causes were unconfounded and apparently exerted independent influences on the effect, uncertainty was relatively low. This pattern of results was well captured by a Bayesian causal model, with a couple of notable exceptions: First, the model predicted that confidence in the existence of a causal link should increase as the interaction became apparent, while human judgments suggested that it decreases. Likewise, the model predicted a decrease in the degree of uncertainty about causal strength as the interaction became apparent, while mean rated levels of uncertainty increase. Thus, while human judgments are consistent with the notion of high uncertainty due to a violation of causal power assumptions, the Bayesian causal model suggests that uncertainty in strength estimates will decrease with the opportunity to observe a candidate cause without its compound counterpart, even when those observations are inconsistent with the assumption of independent causal influence. Notably, the ordinal predictions of the model were robust across several variations, including using a linear rather than a noisy-OR integration function (Lu et al., 2008), using necessary and sufficient rather than uniform priors (Lu et al., 2008), and using a weighted average across graphs (Meder et al., 2014), rather than the most likely graph, to model strength estimates.

Although the Bayesian causal model accurately predicts high uncertainty when there is perfect confounding (i.e., when causes always occur in combination), it also predicts high uncertainty for other reasons, such as when variance is high or samples are small (Griffiths and Tenenbaum, 2005). In contrast, according to the causal power view, confounding constitutes a unique source of uncertainty—a violation of the boundary conditions for causal inference—that is presumably impervious to any bias-reducing influence of increasing sample size: Unlike uncertainty due to small samples, uncertainty due to confounding cannot be resolved by collecting more data, as long as candidate causes continue to only occur in combination. Confounding, therefore, has distinct implications for exploratory behavior and sampling strategies. As noted, when causes are perfectly confounded, the power-PC equations contain multiple unknowns, so that causal strength is not uniquely defined (see Cheng, 1997). However, while this constitutes a signal specific to violations of causal power assumptions, the power-PC theory does not provide an explicit, quantitative, measure of uncertainty. Meanwhile, the Bayesian causal model does provide explicit representations of uncertainty (in both structure and strength estimates) that vary with violations of causal power assumptions, but does not separate these, quantitatively or qualitatively, from uncertainty due to variance or small samples. Further work is needed to determine if accounting for human causal inference warrants a model that provides both a categorical signal specific to violations of causal power assumptions and a continuous measure of corollary uncertainty.

The results also suggest that a violation of the assumption of independent causal influence generates relatively high levels of uncertainty. Importantly, at the end of the second phase of the study, participants were asked to provide judgments about the target cause after many observations of that cause in the absence of its compound counterpart. Thus, they could have based their judgments solely on such trials, ignoring the instruction to consider all of the information presented and avoiding the confusion arising from inconsistencies across elemental and compound trials. The increase in uncertainty as the interaction was revealed suggests that they did in fact consider all of the trials, as instructed. It is likely, however, that had they been asked to provide judgments about a novel compound, consisting of the target cause and an alternative cause with which it had not been previously paired, a more profound increase in uncertainty would have resulted, as such judgments would require generalization of causal knowledge, a process that depends critically on the assumption of causal invariance: if a cause can no longer be assumed to retain its capacity to influence an effect regardless of the spatial or temporal context, the very basis of generalization has been eliminated (Liljeholm and Cheng, 2007; Liljeholm et al., 2007; Cheng et al., 2013). Consequently, once a candidate cause has been found to violate the default assumption of independent influence, one might expect induction about that cause in novel contexts and compounds to be fraught with uncertainty.

Implications for generalization notwithstanding, a substantial literature has demonstrated that humans and other animals are quite capable of treating compound causes differently from what would be expected based on their individual elements, when contingencies so demand (Holland and Block, 1983; Bellingham et al., 1985; Lachnit and Kimmel, 1993; Shanks et al., 1998; Shanks and Darby, 1998; Young et al., 2000; Lober and Lachnit, 2002; Harris et al., 2008). For example, rodents and humans alike can learn that an effect occurs in the presence of either X or Y, but not in the presence of an XY compound (Lachnit and Kimmel, 1993; Harris et al., 2008). Such behavior can be modeled using a “configural” representational unit that corresponds uniquely to the XY combination; a solution that has been adopted by the conjunctive power-PC theory (Novick and Cheng, 2004), by various connectionist error-reduction models (Pearce, 1987, 1994, 2002; Schmajuk and DiCarlo, 1992) and, indeed, by the Bayesian causal framework (Yuille and Lu, 2008). One might suppose, then, that a Bayesian model that includes graphs in which the combination of the target and alternative cause is uniquely represented by a configural node would provide a better account of judgments in the interaction condition than the model considered here. Note, however, that the presence of a configural node eliminates the variance due to inconsistencies across elemental and compound trials that would otherwise be attributed to elemental nodes, and that is the source of the associated uncertainty. Consequently, the inclusion of graphs with a configural node should decrease, rather than increase, the model-derived uncertainty about the influence of the target cause, resulting in an even poorer fit to the data presented here.

An alternative approach to causal interactions, recently proposed by Lucas and Griffiths (2010), is to specify a conjunctive integration function, or “functional form.” For example, a function may specify that at least two causes must be present to generate the effect. Inferences about causal structure can then be drawn across multiple possible functional forms, potentially with different priors (Lucas and Griffiths, 2010; Lucas et al., 2014). Although priors that favor independent over conjunctive functional forms may yield a pattern of results similar to that observed here, it should be noted that the approach advocated by Lucas and colleagues differs profoundly from the principles outlined by theories on causal power. First according to Lucas and Griffiths (2010), learning of functional forms is strictly domain-specific, such that generalization of a particular functional form, based on an increase in its priors due to previous experience, pertains only to the specific causal mechanism for which the form has been observed to apply. In contrast, the causal power view specifies a set of domain-general constraints that enable causal discoveries from statistical regularities regardless of specific causal mechanisms. Second, whereas according to Lucas and Griffiths the appropriateness of a given functional form, be it linear, noisy-OR, or conjunctive, depends strictly on its observed applicability within a particular domain, the causal power approach (Cartwright, 1989; Cheng, 1997) emphasizes that only those forms that follow from the assumption of independent causal influence, such as the noisy-OR, enable coherent transfer of causal knowledge.

In summary, this study assessed how violations of boundary conditions for computing causal power influenced uncertainty in causal judgments. Consistent with previous work showing sensitivity to confounding in exploratory behavior, estimates of outcome probabilities, and causal model selection (Kushnir and Gopnik, 2005; Meder et al., 2006; Schulz and Bonawitz, 2007; Schulz et al., 2007), confounding was found to modulate uncertainty about the existence and strength of a causal relationship. Likewise, uncertainty about the existence and strength of a causal relationship was greater when causes interacted than when they appeared to exert their influences independently. Future work may be aimed at assessing how these distinct sources of uncertainty influence the selection of contingency information and the generalization of causal knowledge.

### Conflict of interest statement

The author declares that the research was conducted in the absence of any commercial or financial relationships that could be construed as a potential conflict of interest.

## Footnotes

^1^Note that “independent causal influence” is a defeasible assumption that can be revised in the face of counterevidence.

^2^The mnemonic labels C and I, indicating “confounded” and “interacting” causes respectively, are used here to facilitate the presentation of the design. In the actual study, to avoid any bias, candidate causes were arbitrary labeled X, Y, P, and Q, with the assignment of labels to conditions being counterbalanced across participants.

^3^Integrals were numerically integrated.

## Appendix

### A Bayesian model of structure and strength judgments

As in Griffiths and Tenenbaum's (2005) causal support model, support for a given target cause, C, is defined here as the log likelihood ratio in favor of graphs in which there is a link between that cause and the effect (Graph1 and Graph3), over graphs in which there is no such link (Graph0 and Graph2).

(A1) $$\underset{C\to E}{\sup}=\log\frac{P(D|Graph1)+P(D|Graph3)}{P(D|Graph0)+P(D|Graph2)}.$$

Each graph has a set of parameters θ, which are the causal strengths *w* of the background (*w*_*B*_), target candidate cause (*w*_*C*_), and alternative candidate cause (*w*_*A*_), respectively (i.e., *w*_*B*_ only in Graph0, *w*_*B*_ and *w*_*C*_ in Graph1, *w*_*B*_ and *w*_*A*_ in Graph2, and *w*_*B*_, *w*_*C*_ and *w*_*A*_ in Graph3). The likelihoods, P(D|Graph_*i*_), are obtained by averaging out the relevant unknown parameters, such that, for Graph0

(A2) $$P(D|Graph0)=\int_{0}^{1}P(D|w_{B},Graph0)P(w_{B}|Graph0)dw_{B},$$

for Graph1

(A3) $$\begin{array}{l} P(D|Graph1)=\int_{0}^{1}\int_{0}^{1}P(D|w_{B},w_{C},Graph1)\\ \;P(w_{B},w_{C}|Graph1)dw_{B}dw_{C}, \end{array}$$

for Graph2

(A4) $$\begin{array}{l} P(D|Graph2)=\int_{0}^{1}\int_{0}^{1}P(D|w_{B},w_{A},Graph2)\\ \;P(w_{B},w_{A}|Graph2)dw_{B}dw_{A}, \end{array}$$

and for Graph3

(A5) $$\begin{array}{l} P(D|Graph3)=\int_{0}^{1}\int_{0}^{1}\int_{0}^{1}P(D|w_{B},w_{C},w_{A},Graph3)\\ \;P(w_{B},w_{C},w_{A}|Graph3)dw_{B}dw_{C}dw_{A}, \end{array}$$

where *P(*θ*|Graph*_*i*_*)* denotes the priors on causal strength parameters, which are assigned independent uniform, Beta(1,1), distributions. The likelihoods *P(D|*θ*, Graph*_*i*_*)* are computed using the noisy-OR parameterization (Pearl, 1988), for which causal power (Cheng, 1997) is the maximum likelihood estimate (Griffiths and Tenenbaum, 2005).

Summarizing data D by contingencies *N(e,c,a)*, the frequencies of each combination of the presence versus absence of the effect, target cause and alternative cause, the likelihood term for Graph0 (setting *w*_*C*_ = 0 and *w*_*A*_ = 0) is

(A6) $$\begin{array}{l} P\left(D|w_{B},Graph0\right)=\left(\begin{array}{c} N\left(c^{-},a^{-}\right)\\ N\left(e^{+},c^{-},a^{-}\right) \end{array}\right)\times \left(\begin{array}{c} N\left(c^{+},a^{-}\right)\\ N\left(e^{+},c^{+},a^{-}\right) \end{array}\right)\\ \;\times \left(\begin{array}{c} N\left(c^{-},a^{+}\right)\\ N\left(e^{+},c^{-},a^{+}\right) \end{array}\right)\times \left(\begin{array}{c} N\left(c^{+},a^{+}\right)\\ N\left(e^{+},c^{+},a^{+}\right) \end{array}\right)\\ \;w_{B}^{N\left(e^{+},c^{-},a^{-}\right)+N\left(e^{+},c^{+},a^{-}\right)+N\left(e^{+},c^{-},a^{+}\right)+}\\ \;N{\left(e^{+},c^{+},a^{+}\right)}_{\left(1-w_{B}\right)}N\left(e^{-},c^{-},a^{-}\right)+\\ \;N\left(e^{-},c^{+},a^{-}\right)+N\left(e^{-},c^{-},a^{+}\right)+N\left(e^{-},c^{+},a^{+}\right), \end{array}$$

where (nk) denotes the number of ways of picking *k* unordered outcomes from *n* possibilities. *N(c*^+^*)* indicates the frequency of events in which the target cause is present, with analogous definitions for the other *N(.)* terms. To improve legibility, in Equations (A7–A9) below, the four (nk) terms, which are identical to those in Equation (A6), have been omitted. Moreover, *N(.)* terms reflect marginalization over causes for which no link exists in the relevant graph, such that, for example, the term “*N(e*^+^*, c*^+^*)”* in Equation (A7) indicates the frequency of events in which both the effect and target cause are present, summed across the presence and absence of the alternative candidate cause.

Thus, the likelihood term for Graph1 (setting *w*_*A*_ = 0) is

(A7) $$\begin{array}{l} P\left(D|w_{B},w_{C},Graph1\right)=w_{B}^{N\left(e^{+},c^{-}\right)}{\left(1-w_{B}\right)}^{N\left(e^{-},c^{-}\right)}\\ \;{\left[w_{B}+w_{C}-w_{B}w_{C}\right]}^{N\left(e^{+},c^{+}\right)}\\ \;{\left[1-w_{B}-w_{C}+w_{B}w_{C}\right]}^{N\left(e^{-},c^{+}\right)}, \end{array}$$

that for Graph2 (setting *w*_*C*_ = 0) is

(A8) $$\begin{array}{l} P\left(D|w_{B},w_{A},Graph2\right)=w_{B}^{N\left(e^{+},a^{-}\right)}{\left(1-w_{B}\right)}^{N\left(e^{-},a^{-}\right)}\\ \;{\left[w_{B}+w_{A}-w_{B}w_{A}\right]}^{N\left(e^{+},a^{+}\right)}\\ \;{\left[1-w_{B}-w_{A}+w_{B}w_{A}\right]}^{N\left(e^{-},a^{+}\right)}, \end{array}$$

and that for Graph3 is

(A9) $$\begin{array}{l} P\left(D|w_{B},w_{C},w_{A},Graph3\right)=w_{B}^{N\left(e^{+},c^{-},a^{-}\right)}{\left(1-w_{B}\right)}^{N\left(e^{-},c^{-},a^{-}\right)}\\ {\left[w_{B}+w_{C}-w_{B}w_{C}\right]}^{N\left(e^{+},c^{+},a^{-}\right)}{\left[1-w_{B}-w_{C}+w_{B}w_{C}\right]}^{N\left(e^{-},c^{+},a^{-}\right)}\\ {\left[w_{B}+w_{A}-w_{B}w_{A}\right]}^{N\left(e^{+},c^{-},a^{+}\right)}{\left[1-w_{B}-w_{A}+w_{B}w_{A}\right]}^{N\left(e^{-},c^{-},a^{+}\right)}\\ {\left[w_{B}+w_{C}+w_{A}-w_{B}w_{C}-w_{B}w_{A}-w_{C}w_{A}+w_{B}w_{C}w_{A}\right]}^{N\left(e^{+},c^{+},a^{+}\right)}\\ {\left[1-w_{B}-w_{C}-w_{A}+w_{B}w_{C}+w_{B}w_{A}+w_{C}w_{A}-w_{B}w_{C}w_{A}\right]}^{N\left(e^{-},c^{+},a^{+}\right)}. \end{array}$$

Causal strength estimates were modeled as the mean of the posterior distribution *P(w*_*C*_*|D)* from the most likely graph in which a link existed between the target cause and the effect (i.e., either Graph1 or Graph3 depending on which had the highest likelihood). The posterior distribution is obtained by applying Bayes' rule, such that, if for example Graph3 had the highest likelihood,

(A10) $$\begin{array}{l} P(w_{C}|D,Graph3)\\ =\int_{0}^{1}\int_{0}^{1}\frac{P(D|w_{B},w_{C},w_{A},Graph3)P(w_{B},w_{C},w_{A}|Graph3)}{P\left(D\right)}dw_{B}dw_{A}, \end{array}$$

where *P(D|w*_*B*_*,w*_*C*_*,w*_*A*_*,Graph3)* is the likelihood term [see Equation (A9)]. *P(w*_*B*_*,w*_*C*_*,w*_*A*_*|Graph3)* refers to the prior probabilities of causal strength parameters, again set to independent *Beta(1,1)* distributions, and *P(D)* is the normalizing term, denoting the probability of the observed data. The mean of *w*_*C*_ is given by

(A11) $${\bar{w}}_{C}=\int_{0}^{1}w_{C}P(w_{C}|D)dw_{C.}$$

Finally, uncertainty about strength is modeled as the Shannon entropy *H(w*_*c*_*)* of *P(w*_*C*_*|D)*

(A12) $$H(w_{C})=-\int_{0}^{1}P(w_{C}|D)\ln P(w_{C}|D)dw_{C}.$$

MATLAB code implementing the model using numerical integration is available in the online supplementary materials.
